# Autoimmune gastritis diverges from a shared corpus origin: molecular reconstruction of the adenocarcinoma axis and clinical anchoring of the neuroendocrine axis

**DOI:** 10.3389/fimmu.2026.1899802

**Published:** 2026-08-25

**Authors:** Jingna Tao, Linghan Meng, Xiyan Zhang, Liju Zhang, Tong Li, Zhihong Li

**Affiliations:** 1 Dongzhimen Hospital, Beijing University of Chinese Medicine, Beijing, China; 2 Department of Integrated Traditional Chinese and Western Medicine Oncology, Hangzhou Cancer Hospital, Hangzhou, China

**Keywords:** autoimmune gastritis, enterochromaffin-like cell, gastric neuroendocrine tumour, hypergastrinaemia, intestinal metaplasia, precancerous progression, single-cell RNA sequencing, spasmolytic polypeptide-expressing metaplasia

## Abstract

Autoimmune gastritis (AIG) arises from a T-cell–mediated immune attack on the gastric parietal cell and carries a dual neoplastic risk: gastric adenocarcinoma through spasmolytic polypeptide-expressing metaplasia (SPEM) and intestinal metaplasia, and type-1 gastric neuroendocrine tumour (NET) through hypergastrinaemia-driven enterochromaffin-like (ECL) cell hyperplasia. This “one origin, two fates” framework is conceptually established but has never been given a molecular trajectory from an AIG origin, and molecular data for the neuroendocrine endpoint are essentially absent. We integrated public single-cell transcriptomes of the human AIG corpus (GSE271866; three samples, 14,323 cells) and of a histologically staged premalignant cascade (GSE134520; 44,012 cells) with a cross-sectional cohort of approximately 203 AIG patients, treating the two axes asymmetrically: the adenocarcinoma axis was reconstructed at the molecular level, whereas the neuroendocrine axis was clinically and literature anchored. The AIG corpus showed near-absent parietal cells (0.45%) with extensive SPEM (18.6%) and intestinal metaplasia (16.3%). Cascade pseudotime increased monotonically with histological stage (Spearman’s *ρ* = 0.496; unchanged on the unbridged graph) and defined a gastric-to-intestinal progression signature that, transferred to the AIG corpus, tracked the AIG-intrinsic pseudotime (*ρ* = 0.673). Most AIG metaplastic cells were projected to the strong-metaplasia stage (90.9%; bootstrap 95% CI 84–98%; *n* = 55), a suggestive rather than definitive placement that is nonetheless consistent with AIG occupying the atrophy-to-metaplasia segment of the adenocarcinoma axis. In the cohort, hypergastrinaemia scaled with corpus atrophy (gastrin-17 versus pepsinogen I/II ratio *ρ* = −0.413, *p* = 9.9 × 10^−8^), and single-cell analysis confirmed a gastrin-responsive ECL population (*HDC*^+^ 93%, *CCKBR*^+^ 76%) whose neuroendocrine programme was orthogonal to the intestinal programme of the adenocarcinoma arm. Because AIG is a prototypic autoimmune disease, we frame how the autoimmune infiltrate steers each fate as the central open immunological question. AIG thus enters the metaplasia-to-cancer axis from a corpus, parietal-cell-loss origin and diverges, through a serum-gastrin–ECL bridge, towards the neuroendocrine fate; we provide an interpretable progression signature and a severity-anchored, hypothesis-generating framework—not an outcome predictor, since the cohort is cross-sectional with essentially no neoplastic endpoints—and report the molecular data gap for the neuroendocrine endpoint as a field-level priority.

## 1 Introduction

Autoimmune gastritis (AIG) is a corpus-restricted chronic gastritis driven by an immune attack on the gastric parietal cell and its H^+^/K^+^-ATPase. The progressive loss of parietal cells produces oxyntic atrophy, achlorhydria, impaired intrinsic-factor secretion, and, classically, pernicious anaemia, whereas the loss of acid-mediated feedback drives a compensatory rise in serum gastrin (1–3). Once regarded as rare, AIG is increasingly recognised, and its long-term significance lies less in its symptomatic burden than in its neoplastic potential (3, 4).

That neoplastic potential is distinctive because it is *dual*. The atrophic, metaplastic corpus mucosa is a substrate for gastric adenocarcinoma arising through the metaplastic sequence of spasmolytic polypeptide-expressing metaplasia (SPEM) and intestinal metaplasia (IM); at the same time, the sustained hypergastrinaemia of achlorhydria is a trophic stimulus for enterochromaffin-like (ECL) cells, predisposing to ECL hyperplasia and type-1 gastric neuroendocrine tumours (NET) (5–7). AIG thus offers a striking example of “one origin, two fates”: a single autoimmune insult to the parietal cell that can diverge towards two biologically unrelated neoplasms. Critically, AIG is a prototypic T-cell–mediated autoimmune disease (8), so the same immune attack that ablates parietal cells also shapes the microenvironment in which these precancerous programmes unfold; how immunity drives progression along either fate remains largely unmapped.

The glandular (adenocarcinoma) arm of this divergence has a mature cell-biological literature. SPEM and IM lineages, their markers (*MUC6*, *TFF2*, and *AQP5* for SPEM; *CDX2*, *MUC2*, and *TFF3* for IM), and their standing in the preneoplastic process have been dissected in detail, increasingly at single-cell and spatial resolution (9–15). Yet, this work has been built overwhelmingly around *Helicobacter pylori*-driven, antrum-predominant injury and the classical Correa cascade (16, 17); the metaplasia-to-cancer trajectory has rarely been reconstructed *from an AIG, corpus, parietal-cell-loss origin*, and almost never anchored to a real AIG patient cohort.

Four gaps follow. First, although the conceptual framework of two distinct gastric-cancer aetiologies (infection versus autoimmunity) is established, it has remained at the level of review and has not been given a molecular trajectory rooted in AIG (5). Second, the clinical risk and staging tools for atrophic gastritis—OLGA staging, the corpus-predominant gastritis index, and recent ACG/AGA management updates—are purely histological or serological and have never been connected to a molecular progression programme (1, 2, 4, 18). Third, the neuroendocrine arm suffers from a near-total *molecular data gap*: beyond clinical and chromogranin-based descriptions of carcinoids and hypergastrinaemia, there is essentially no public expression profile of AIG-related ECL hyperplasia or type-1 gastric NET (6, 7, 19). Fourth, and methodologically, existing AIG transcriptomic studies (including our own) have been framed as *diagnostic discrimination*—telling AIG apart from *H. pylori*-associated atrophic gastritis—and have not addressed *progression or risk*: the question shifts from “what is it?” to “where is it going?”.

Here, we address these gaps with an explicitly *asymmetric dual-axis* design. Using public single-cell transcriptomes (20), we (i) build a single-cell map of the human AIG corpus that fixes the parietal-cell-loss origin and its incipient metaplastic and endocrine programmes; (ii) reconstruct a fully resolved molecular pseudotime of the histological premalignant cascade and distil an interpretable progression signature; (iii) show by reference-based transfer that the AIG corpus epithelium converges onto the metaplasia-to-cancer axis from its corpus origin; (iv) establish the divergent neuroendocrine fate through a serum-gastrin–ECL bridge that exploits a unique clinical cohort, and report the NET molecular data gap itself as a finding; and (v) delineate, honestly, where the immune microenvironment does and does not support a progression mechanism. Throughout, the study is framed as precancerous progression-risk biology, is anchored to a cross-sectional AIG cohort as a severity reference rather than an outcome predictor, and is kept strictly distinct from prior diagnostic work.

## 2 Materials and methods

### 2.1 Study design and rationale

**Figure 1:**
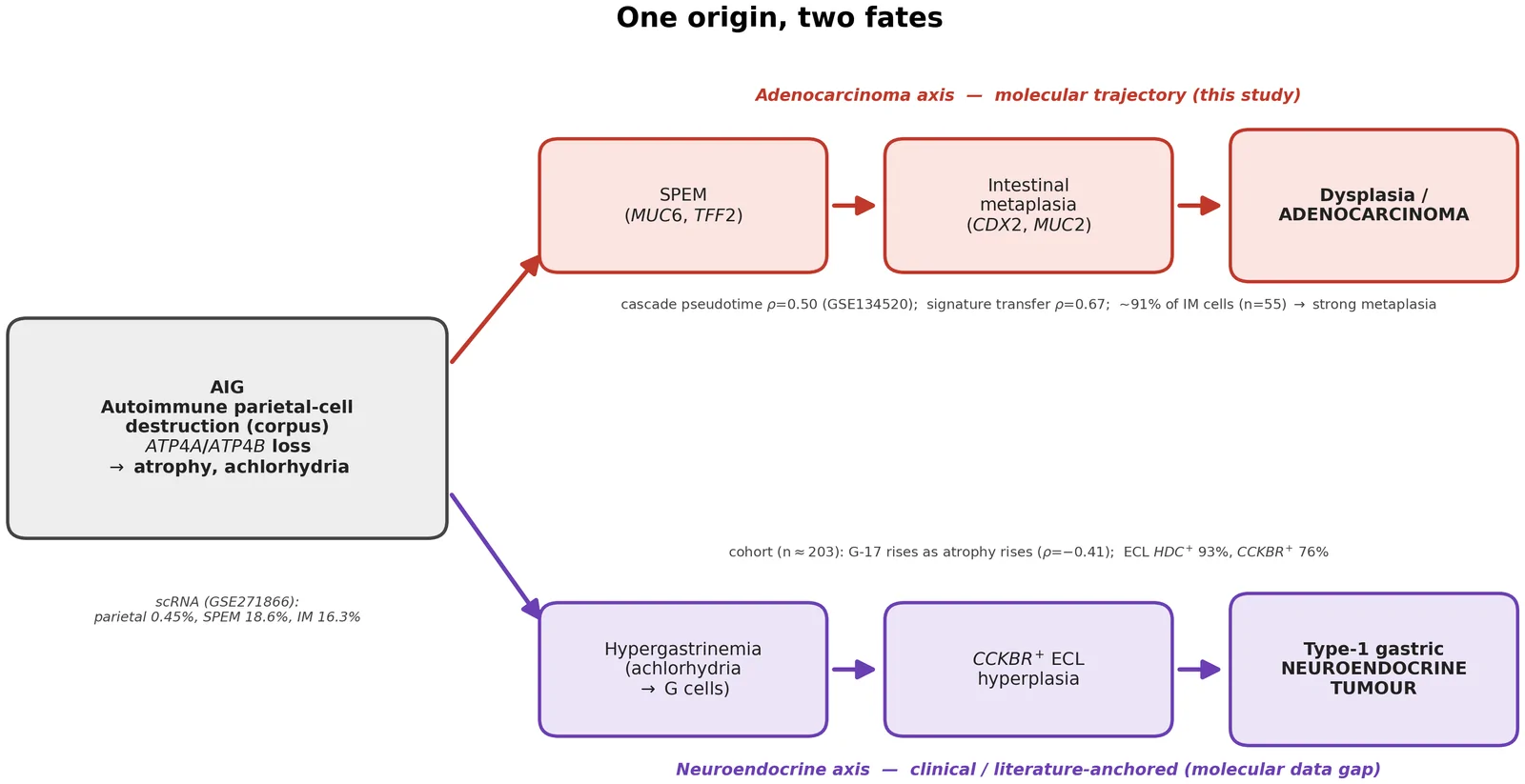
Study concept and asymmetric dual-axis design. Autoimmune destruction of parietal cells (corpus; *ATP4A*/*ATP4B* loss) produces atrophy and achlorhydria, which branches into (i) an adenocarcinoma axis via SPEM (*MUC6*/*TFF2*) and intestinal metaplasia (*CDX2*/*MUC2*) towards dysplasia/adenocarcinoma, reconstructed here as a molecular trajectory; and (ii) a neuroendocrine axis via achlorhydria-driven hypergastrinaemia and *CCKBR*^+^ ECL hyperplasia towards type-1 gastric NET, anchored clinically and in the literature (molecular data gap). Key anchoring statistics from GSE271866, GSE134520, and the clinical cohort are annotated.

We set out to test whether autoimmune gastritis (AIG) feeds a shared, corpus-restricted precancerous programme that subsequently *diverges* into two distinct fates: a glandular (adenocarcinoma) axis proceeding through spasmolytic polypeptide-expressing metaplasia (SPEM) and intestinal metaplasia (IM), and a neuroendocrine axis proceeding through hypergastrinaemia-driven enterochromaffin-like (ECL) cell hyperplasia towards type-1 gastric neuroendocrine tumours (NET). Because publicly available molecular data for the neuroendocrine tumour endpoint are effectively absent (Section 2.9), the design is deliberately *asymmetric*: the adenocarcinoma axis is reconstructed at the molecular level from public single-cell data, whereas the neuroendocrine axis is established as a clinically and literature-anchored secondary arm, bridged through serum gastrin and an ECL transcriptional programme (**Figure 1**). The study is explicitly framed as precancerous *progression-risk biology*; it does not attempt hard oncological outcome prediction, for which our cross-sectional cohort is not powered.

### 2.2 Public single-cell RNA-sequencing datasets

Two human gastric single-cell datasets were obtained from the NCBI Gene Expression Omnibus (GEO).

*AIG corpus origin (GSE271866).* Three human AIG corpus samples (AIG1–AIG3) were used to anchor the disease-specific origin. This series also contains non-human (murine *Helicobacter*/treatment) samples, which were removed prior to analysis to avoid a cross-species, mixed-orthologue merge; after quality control, 14,323 human cells remained. GSE271866 has been used in prior diagnostic work on AIG; here, it serves *only* as an origin anchor for trajectory and metaplasia analysis, a distinct question from diagnostic discrimination (Section 2.9).

*Premalignant cascade reference (GSE134520).* A total of 13 primary human gastric mucosal samples spanning non-atrophic gastritis (NAG), chronic atrophic gastritis (CAG), weak and strong intestinal metaplasia (IMW, IMS), and early gastric cancer (EGC) (21) provided the histologically staged cascade against which the AIG origin was projected; 44,012 cells passed quality control. This generic premalignant cascade is predominantly antrum/*H. pylori*-associated; AIG specificity is therefore not assumed from this dataset but is established by anchoring to GSE271866 (Section 3).

### 2.3 Clinical cohort

A cross-sectional, single-centre cohort of approximately 203 patients with AIG was assembled from paired endoscopic and serological records. Endoscopic severity was recorded as the ROM (Royal Melbourne/endoscopic atrophy) stage; pseudopyloric metaplasia (the histological/endoscopic correlate of SPEM) and intestinal metaplasia were noted where visible. We use “pseudopyloric metaplasia” for the histological/endoscopic finding and “SPEM” for the corresponding single-cell state, treating them as the same metaplastic programme observed at different levels of resolution. Serological variables included gastrin-17 (G-17), the pepsinogen I/II (PG I/II) ratio, parietal cell antibody (PCA) titre, vitamin B12, anti-thyroid antibodies (TPOAb, TGAb), and haemoglobin. The cohort is cross-sectional and contains essentially no hard neoplastic endpoints (adenocarcinoma ≈ 0; NET *n* = 1); it is therefore used strictly as a *severity anchor* and not for outcome prediction. Cohort characteristics are summarised in **Table 1**.

**Table 1:** Characteristics of the cross-sectional autoimmune gastritis cohort (∼203 patients) used as a precancerous severity anchor.

Domain	Variable	Value
Endoscopy	Endoscopic atrophy (ROM) stage 1	79
	ROM stage 2	36
	ROM stage 3	80
	Very-early/indeterminate	8
Histology	Pseudopyloric (SPEM-like) metaplasia	118/160 (∼74%)
	Intestinal metaplasia (endoscopically visible)	11/203 (∼5%)
Serology	Gastrin-17, median (IQR), pmol/L	15.6 (5.0–42.2), *n* = 156
	range; % *>*7; % *>*30	1–1184; 66.7%; 29.5%
	Pepsinogen I/II ratio, median	2.55, *n* = 181
	Parietal cell antibody titre	*n* = 181
	Vitamin B12	*n* = 160
	Haemoglobin	*n* = 105
	TPOAb/TGAb (thyroid comorbidity)	*n* = 104/*n* = 99
Endpoints	Incident adenocarcinoma	≈0
	Incident type-1 gastric NET	1

Denominators differ because not every assay was available for every patient.

### 2.4 Single-cell preprocessing, integration, and annotation

All single-cell analyses used Scanpy (22). Cells were retained with at least 200 detected genes and a mitochondrial transcript fraction below 20%. Counts were total-count normalised and log1p-transformed; the top 2,000 highly variable genes were selected for principal component analysis (30 components). Sample-level batch effects were corrected with Harmony (23), and clusters were identified with the Leiden algorithm (igraph backend) (24) on the Harmony-corrected neighbour graph; UMAP was used for visualisation.

Cell-type annotation followed a *compartment-first* strategy to avoid the mislabelling of rare and transcriptionally subtle epithelial states. Each cluster was first assigned to the epithelial (*EPCAM*, *KRT8/18/19*, and *CDH1*), immune (*PTPRC*), or stromal (*DCN*, *PECAM1*, *VWF*, *COL1A1*, and *PDGFRB*) compartment by the highest mean compartment score. Within each compartment, a higher-resolution Leiden partition (resolution 2.0) was sub-typed by the argmax of lineage marker-gene scores: parietal (*ATP4A* and *ATP4B*), chief (*PGC* and *LIPF*), neck/SPEM (*MUC6*, *TFF2*, and *AQP5*), foveolar (*MUC5AC*, *TFF1*, and *GKN1*), isthmus/stem (*MKI67* and *OLFM4*), intestinal metaplasia (*CDX2*, *MUC2*, and *TFF3*), and enteroendocrine/ECL (*CHGA*, *CHGB*, and *HDC*). Because parietal and ECL cells are rare and are diluted by cluster-level averaging, their abundance was additionally quantified as the *fraction of marker-positive cells*, which recovers rare states that cluster argmax misses.

### 2.5 Trajectory inference and the progression signature

Pseudotime was computed on the epithelial subset using the diffusion-map/diffusion pseudotime (DPT) framework (25). The root was chosen in a *data-driven* manner as the epithelial cluster with the lowest mean histological stage ordinal (cascade reference) or, for the AIG corpus, the isthmus/stem cluster, consistent with a regenerative origin. Where the *k*-nearest-neighbour graph contained disconnected components that left pseudotime undefined (infinite) for an otherwise valid lineage, a minimal set of bridging edges between the nearest cells of adjacent components was added so that pseudotime was finite for 100% of cells; this pragmatic repair is reported transparently and conclusions are framed as state coexistence and thematic molecular ordering rather than strict lineage kinetics or causality. In practice, bridging was required only for the AIG corpus graph; the cascade (GSE134520) epithelial graph was already fully connected, and a sensitivity analysis that recomputed cascade pseudotime without any bridging gave an essentially identical stage association (Section 3.2), confirming that the ordering is not a bridging artefact.

An interpretable *progression signature* was defined on the cascade reference (GSE134520) as the genes whose log-normalised expression correlated most strongly (Pearson) with epithelial pseudotime, split into an up-regulated module (positively correlated) and a down-regulated module (negatively correlated). The full ranked lists are provided in **Table 2**.

**Table 2:** The pseudotime-derived progression signature on the premalignant cascade (GSE134520).

Up-regulated (intestinal)			Down-regulated (gastric identity)		
Rank	Gene	*r*	Rank	Gene	*r*
1	*REG4*	0.69	1	*MUC5AC*	−0.79
2	*PRAP1*	0.66	2	*GKN2*	−0.76
3	*FABP2*	0.66	3	*TFF2*	−0.70
4	*FABP1*	0.66	4	*GKN1*	−0.66
5	*ANPEP*	0.63	5	*S100P*	−0.61
6	*TM4SF20*	0.59	6	*PGC*	−0.49
7	*ALDOB*	0.58	7	*PSCA*	−0.44
8	*TMPRSS15*	0.51	8	*MSMB*	−0.35
9	*RBP2*	0.51	9	*LYZ*	−0.30
10	*KRT20*	0.51	10	*MT1G*	−0.30
11	*IL32*	0.49	11	*PSAPL1*	−0.29
12	*ADIRF*	0.46	12	*SOSTDC1*	−0.26
13	*ZG16*	0.46	13	*MT1X*	−0.25
14	*PCK1*	0.45	14	*FOS*	−0.24
15	*TFF3*	0.44	15	*DEFB1*	−0.21

Genes are ranked by Pearson correlation *r* between log-normalised expression and epithelial pseudotime. The up-regulated module marks an intestinal differentiation/metaplasia programme; the down-regulated module marks loss of gastric/foveolar identity.

### 2.6 Cross-dataset signature transfer and label projection

To test whether the AIG corpus converges onto the cascade, the progression signature was transferred to the AIG cells rather than merging the two datasets (which would confound disease with batch). Two complementary, reference-based approaches were used. First, a per-cell progression score was computed in the AIG data with Scanpy score_genes as the difference between the up- and down-module scores (prog_score = score_↑_ −score_↓_), using 24 up- and 25 down-module genes present in both datasets, and correlated with the AIG-intrinsic pseudotime. Second, Scanpy ingest projected AIG epithelial cells onto the cascade reference embedding to predict, for each AIG cell, its most likely cascade stage (shared genes scaled to unit variance, 30 principal components, and the standard reference *k*-NN graph; the robustness of the small AIG intestinal-metaplasia projection, *n* = 55, was quantified by 2,000-fold bootstrap resampling). Both are feature/label-transfer methods; results are reported as relative orderings and predicted-stage proportions, not as a joint embedding or as absolute expression levels.

### 2.7 Divergent-fate programme scoring and ECL characterisation

Two fate programmes were scored per cell with score_genes: an *intestinal* programme (*CDX2*, *MUC2*, *TFF3*, *FABP1*, *FABP2*, *REG4*, and *KRT20*) and a *neuroendocrine* programme (*CHGA*, *CHGB*, *HDC*, *CCKBR*, *GAST*, *SCG2*, *NEUROD1*, and *ASCL1*). To confirm the identity and gastrin responsiveness of the ECL population, marker-positive fractions were computed for ECL versus all other cells, with particular attention to *HDC* (histidine decarboxylase, ECL-specific), *CCKBR* (the gastrin/CCK-B receptor), and *GAST* (gastrin itself, expected to be negative in ECL cells).

### 2.8 Immune microenvironment analysis (exploratory)

As an exploratory mechanistic arm, immune compartment fractions were tabulated by cascade stage, and cytotoxic (*CD8A*, *GZMB*, *GZMK*, *NKG7*, *PRF1*, and *GNLY*) and interferon-*γ* response (*STAT1*, *IRF1*, *GBP1/2*, *CXCL9/10*, *HLA-DRA/DRB1*, *B2M*, *TAP1*, *IDO1*, *WARS*, and *PSMB9*) programmes were scored. Associations with progression were assessed both at the single-cell level (epithelial IFN-*γ* score versus pseudotime) and at the sample level (Spearman correlations across the 13 cascade samples). This arm was pre-specified as exploratory because no ligand–receptor inference tool (e.g., CellChat) was applied and the sample-level analysis is underpowered.

### 2.9 Statistical analysis and study-design principles

Monotonic associations were assessed with Spearman’s *ρ*; two-group comparisons with the Mann–Whitney *U* test; multigroup comparisons with the Kruskal–Wallis test. Tests were two-sided with *α* = 0.05. Where extremely small *p*-values arise from very large single-cell counts, we report the effect size alongside the *p*-value and interpret accordingly.

The analysis is predominantly unsupervised (trajectory inference and correlation) and therefore does not involve classifier training or cross-validation. Two design choices nonetheless guard against confounding and information leakage, following the conservative approach of our prior diagnostic work: (i) the two single-cell datasets were *not* pooled into one supervised feature space; instead, the signature was transferred by reference-based projection, avoiding a disease=batch confound that a naive ComBat (26) merge would introduce; and (ii) should a supervised risk stratifier be built in future, feature selection would be nested within cross-validation. Crucially, this study addresses precancerous *progression* biology and shares no analysis, model, or claim with the prior *diagnostic* discrimination of AIG versus *H. pylori*-associated atrophic gastritis; the only shared input is GSE271866, used here purely as an origin anchor.

Single-cell analyses used Scanpy (v1.12) with anndata, harmonypy, scikit-learn, statsmodels, and SciPy under Python 3; random seeds were fixed (bootstrap seed 0) for reproducibility. The code to reproduce all figures and statistics is available from the authors on reasonable request.

## 3 Results

Our evidence chain proceeds in four linked steps and one exploratory arm. We first build a single-cell map of the AIG corpus to fix the disease-specific *origin* (Section 3.1); we then reconstruct the histological premalignant *cascade* and distil an interpretable progression signature (Section 3.2); we show that the AIG corpus *converges* onto this cascade (Section 3.3); we establish the divergent *neuroendocrine fate* through a serum-gastrin–ECL bridge (Section 3.4); and we report a weak, non-specific immune association as an exploratory observation (Section 3.5).

### 3.1 A single-cell map of the AIG corpus captures parietal-cell loss and metaplastic reprogramming at the trajectory origin

**Figure 2:**
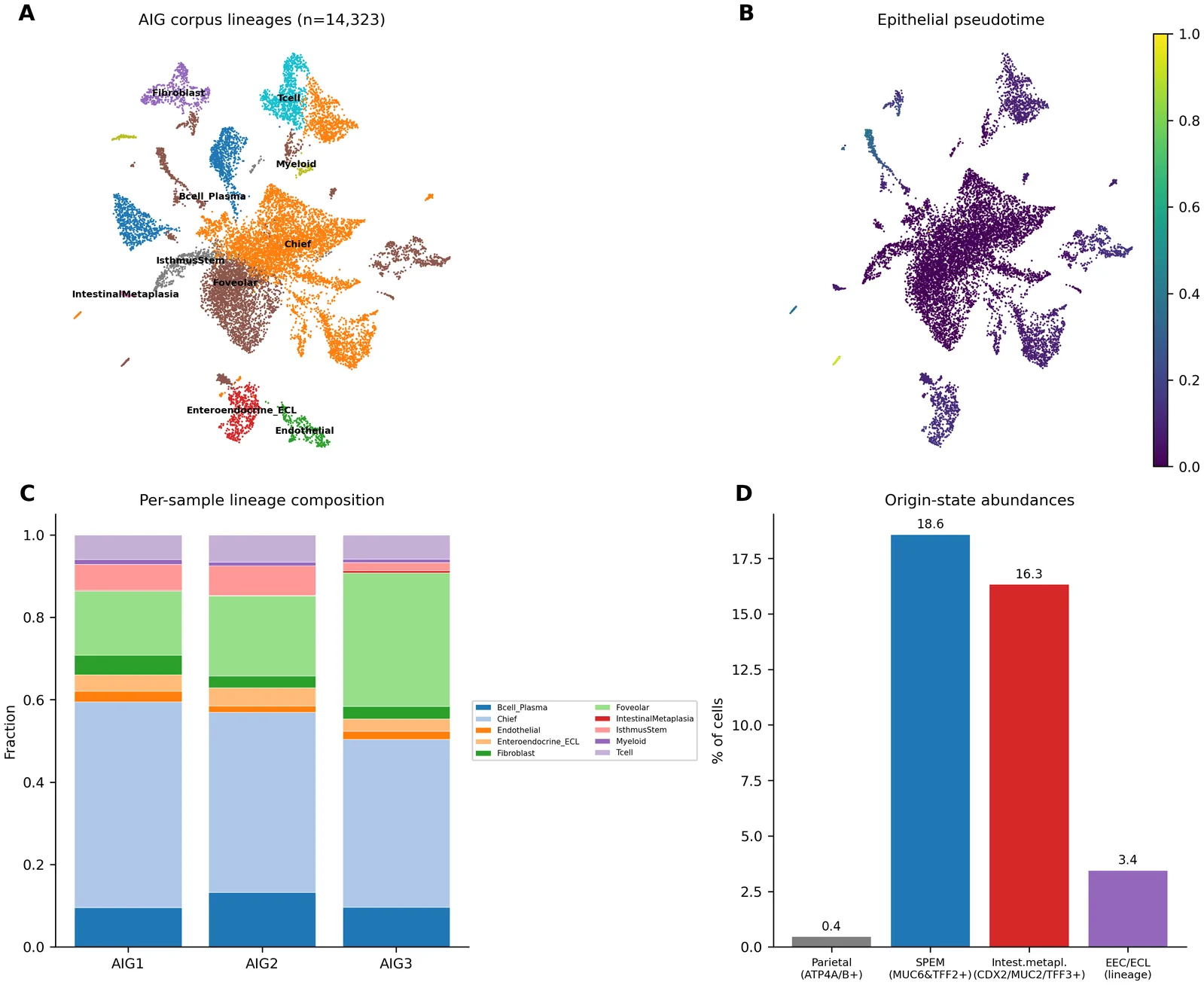
Single-cell map of the AIG corpus origin (GSE271866, 14,323 cells). **(A)** Lineage UMAP with marker-validated annotation. **(B)** Epithelial pseudotime. **(C)** Per-sample lineage composition across the three AIG biopsies. **(D)** Origin-state abundances: parietal cells are near-absent (*ATP4A*/*ATP4B*^+^ 0.45%), whereas SPEM (*MUC6* & *TFF2*^+^ 18.6%), intestinal metaplasia (*CDX2*/*MUC2*/*TFF3*^+^ 16.3%) and an enteroendocrine/ECL population (lineage fraction 3.4%) are present.

Across 14,323 quality-controlled cells from three human AIG corpus biopsies (GSE271866—a small sample that bounds disease-level generalisation and that we revisit as a central limitation—compartment-first annotation resolved the expected epithelial, immune, and stromal lineages, validated by a marker dotplot in which chief (*PGC* and *LIPF*), foveolar (*MUC5AC*, *TFF1*, and *GKN1*), intestinal metaplastic (*MUC2* and *TFF3*), isthmus/stem (*MKI67*), and enteroendocrine (*CHGA* and *CHGB*) markers fell on the diagonal (**Figure 2**).

Three features define the AIG origin state. First, parietal cells were nearly absent: only 0.45% of cells were *ATP4A*/*ATP4B* positive, closely matching the 0.42% reported independently, confirming autoimmune parietal-cell destruction at single-cell resolution. Second, the corpus showed pronounced metaplastic reprogramming: SPEM (co-expression of *MUC6* and *TFF2*) accounted for 18.6% of cells and intestinal metaplasia (*CDX2*/*MUC2*/*TFF3*) for 16.3%, providing the substrate for the adenocarcinoma axis. Third, a distinct enteroendocrine/ECL population was present (3.4% of cells, high *CHGA*/*CHGB*), foreshadowing the neuroendocrine fork. Epithelial pseudotime (finite for all cells after the graph bridging described in Methods) arranged the lineages as a thematic ordering—from a regenerative isthmus/stem root through normal differentiation towards both metaplastic and endocrine states (isthmus/stem → chief → foveolar → intestinal metaplasia →ECL)—which we read as state coexistence rather than measured lineage kinetics. Thus, AIG presents, at its corpus origin, the parietal-cell loss and the two incipient programmes (metaplastic and endocrine) whose divergence the remainder of the study dissects.

### 3.2 A molecular pseudotime reconstructs the histological premalignant cascade and defines an interpretable progression signature

**Figure 3:**
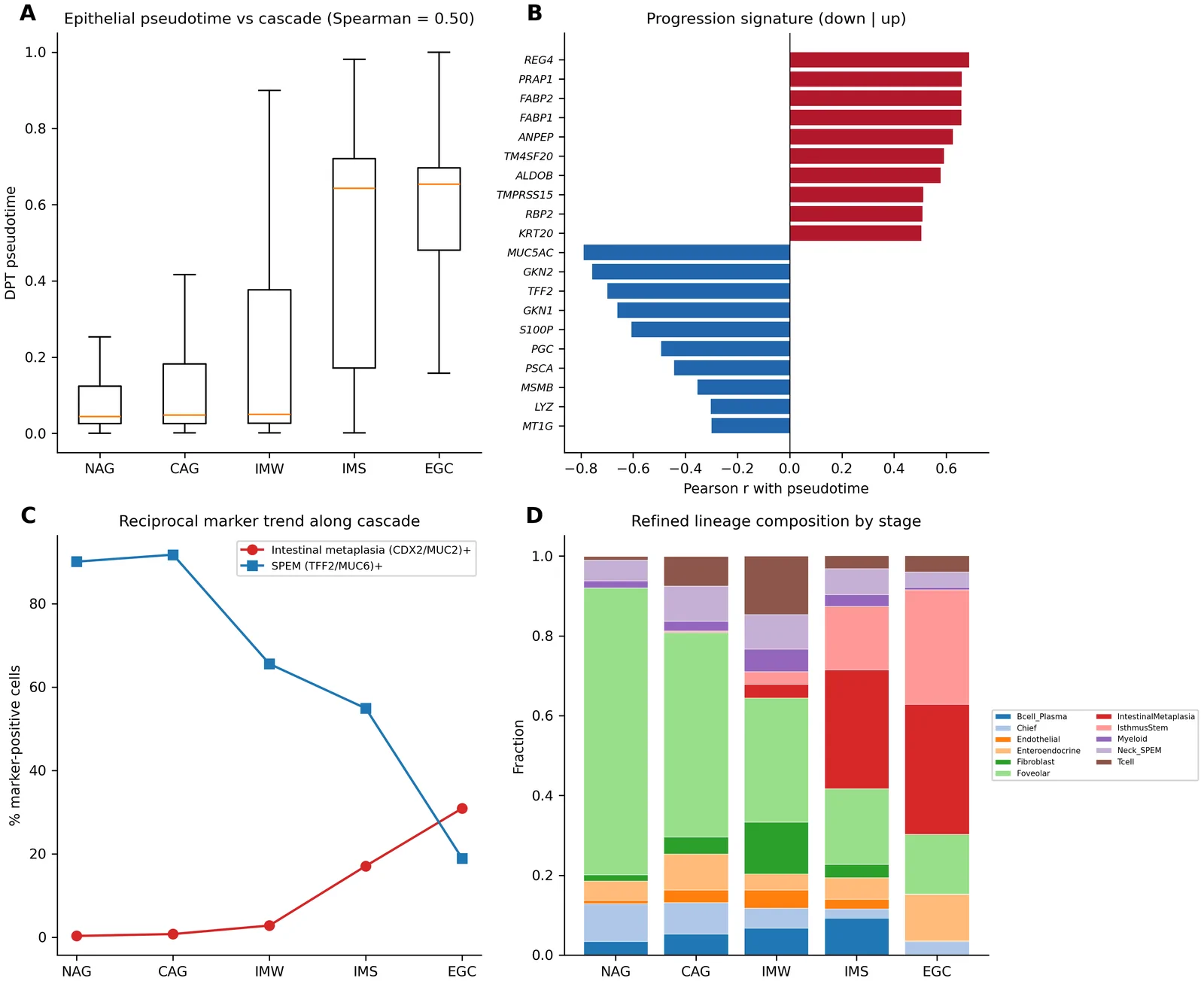
Molecular pseudotime of the premalignant cascade (GSE134520, 44,012 cells). **(A)** Epithelial pseudotime increases monotonically from NAG to EGC (single-cell Spearman’s *ρ* = 0.496 versus stage ordinal). **(B)** Progression signature: genes most negatively (blue, gastric identity) and most positively (red, intestinal) correlated with pseudotime. **(C)** Reciprocal trend of intestinal-metaplasia markers (rising) and SPEM markers (declining) along the cascade. **(D)** Refined lineage composition by stage.

To obtain a fully resolved adenocarcinoma trajectory, we analysed 44,012 cells from 13 primary samples spanning NAG, CAG, IMW, IMS, and EGC (GSE134520) (21). Epithelial pseudotime increased *monotonically* across the histological stages (mean pseudotime: NAG 0.099 *<* CAG 0.143 *<* IMW 0.182 *<* IMS 0.493 *<* EGC 0.580) and at the single-cell level pseudotime tracked the stage ordinal (Spearman’s *ρ* = 0.496; **Figure 3**). Throughout, because single-cell correlations are computed over tens of thousands of cells, we base interpretation on the effect size (*ρ*) and treat the correspondingly minuscule *p*-values as non-informative—they reflect the large cell count rather than the strength of the effect. This ordering was not an artefact of graph manipulation: the epithelial *k*-NN graph was fully connected without any bridging, and recomputing pseudotime on the unbridged graph left the association essentially unchanged (100% finite; Spearman’s *ρ* = 0.491). The molecular trajectory therefore recapitulates the morphological cascade.

The genes most strongly correlated with pseudotime defined a coherent, biologically interpretable progression signature of a gastric-to-intestinal identity switch (**Table 2**). The up-regulated module comprised intestinal differentiation/metaplasia genes (*REG4*, *r* = 0.69; *PRAP1*; *FABP1*/*FABP2*; *ANPEP*; *ALDOB*; *TMPRSS15*; *RBP2*; *KRT20*; *ZG16*), whereas the down-regulated module comprised gastric/foveolar identity genes (*MUC5AC*, *r* = −0.79; *GKN2*; *TFF2*; *GKN1*; *PGC*; *PSCA*). Consistently, marker-positive intestinal-metaplastic cells rose monotonically along the cascade (0.3% → 0.8% → 2.8% → 17.1% → 30.9% from NAG to EGC) whereas SPEM declined (90.1% → 18.9%).

Compartment-first re-annotation at high resolution (31 clusters) partitioned the data into epithelial (34,662), immune (6,374), and stromal (2,976) compartments and fully resolved the epithelial subtypes (neck/SPEM, chief, isthmus/stem, and intestinal metaplasia), correcting an initial coarse label (a putative 36% “myeloid” fraction that was in fact mis-assigned epithelium fell to 3%). The refined stage composition sharpened the same trend: foveolar cells fell from 0.72 (NAG) to 0.15 (EGC), whereas intestinal metaplasia rose from 0.00 to 0.33 and isthmus/stem (proliferative) from 0.00 to 0.29. Because the trajectory, signature, and marker-positive conclusions rest on scores and marker positivity rather than on cluster labels, this refinement increased lineage resolution without altering the core results.

### 3.3 AIG corpus epithelium converges onto the metaplasia-to-cancer axis

**Figure 4:**
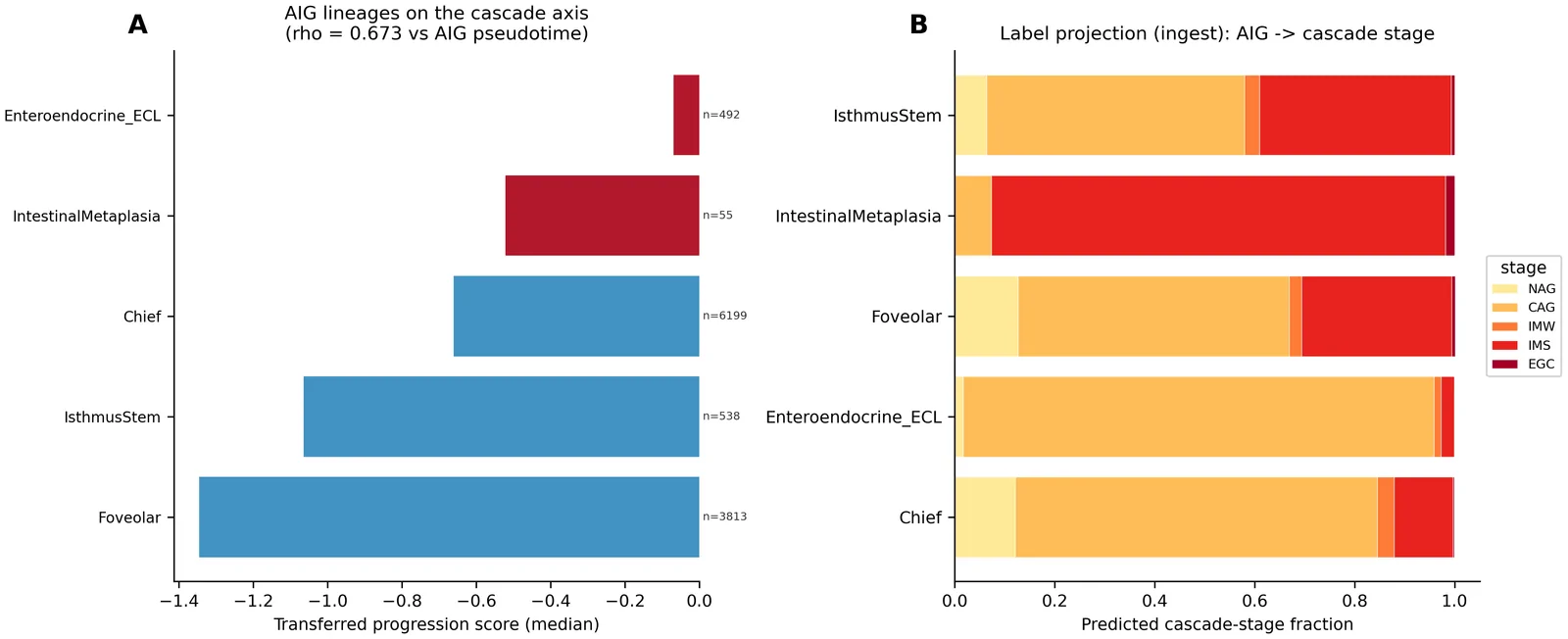
AIG corpus converges onto the metaplasia-to-cancer axis. **(A)** The transferred progression score ranks AIG lineages from normal differentiation (low) to metaplastic/endocrine states (high) and correlates with AIG-intrinsic pseudotime (*ρ* = 0.673); scores are relative to the antrum-derived reference. **(B)** Label projection (ingest) maps most AIG intestinal-metaplastic cells (90.9%; 95% CI 84–98%;*n* = 55, suggestive) to the strong-metaplasia (IMS) stage and the bulk of the epithelium to the atrophic (CAG) stage.

We next asked whether the AIG origin (Section 3.1) lies *on* the cascade (Section 3.2). Transferring the progression signature to the AIG epithelium (24 up- and 25 down-module genes; reference-based scoring, no dataset merge), the AIG per-cell progression score correlated strongly with the AIG-intrinsic pseudotime (Spearman’s *ρ* = 0.673; **Figure 4**), indicating that the internal direction of change in the AIG corpus matches the generic metaplasia-to-cancer direction. Both halves of the signature contributed in the expected directions within the AIG corpus (up-module Spearman’s *ρ* = 0.34, down-module *ρ* = −0.59 versus pseudotime), confirming that the gastric-to-intestinal switch operates inside AIG itself rather than being imposed by the reference.

The score ranked the AIG lineages exactly as the model predicts: normal differentiation scored lowest and metaplastic/endocrine states highest (median progression score: foveolar −1.35 *<* isthmus/stem −1.06 *<* chief −0.66 *<* intestinal metaplasia −0.52 *<* enteroendocrine/ECL −0.07). AIG intestinal-metaplastic cells scored significantly above normally differentiated cells (Mann–Whitney *p* = 1.8×10^−7^; metaplasia *n* = 55 versus normal *n* = 10,012). Independently, label projection (ingest) onto the cascade reference mapped most AIG intestinal-metaplastic cells (90.9%; bootstrap 95% CI 83.6–98.2%) to the strong-metaplasia (IMS) stage, whereas the bulk of the AIG epithelium (chief 72%, ECL 94%, foveolar 54%) was assigned to the atrophic (CAG) stage. Because this projection rests on a small metaplastic subset (*n* = 55), we read it as suggestive rather than definitive; with that caveat, AIG corpus cells occupy the atrophy-to-strong-metaplasia segment of the cascade rather than its normal end, consistent with AIG entering the metaplasia-to-cancer axis from a corpus, parietal-cell–loss origin, distinct from the *H. pylori*/antral Correa origin. Because the AIG corpus scored lower in absolute terms than the antrum-derived reference, these results are interpreted as relative orderings and predicted-stage proportions, not absolute levels.

### 3.4 A serum-gastrin–ECL bridge defines the divergent neuroendocrine fate

**Figure 5:**
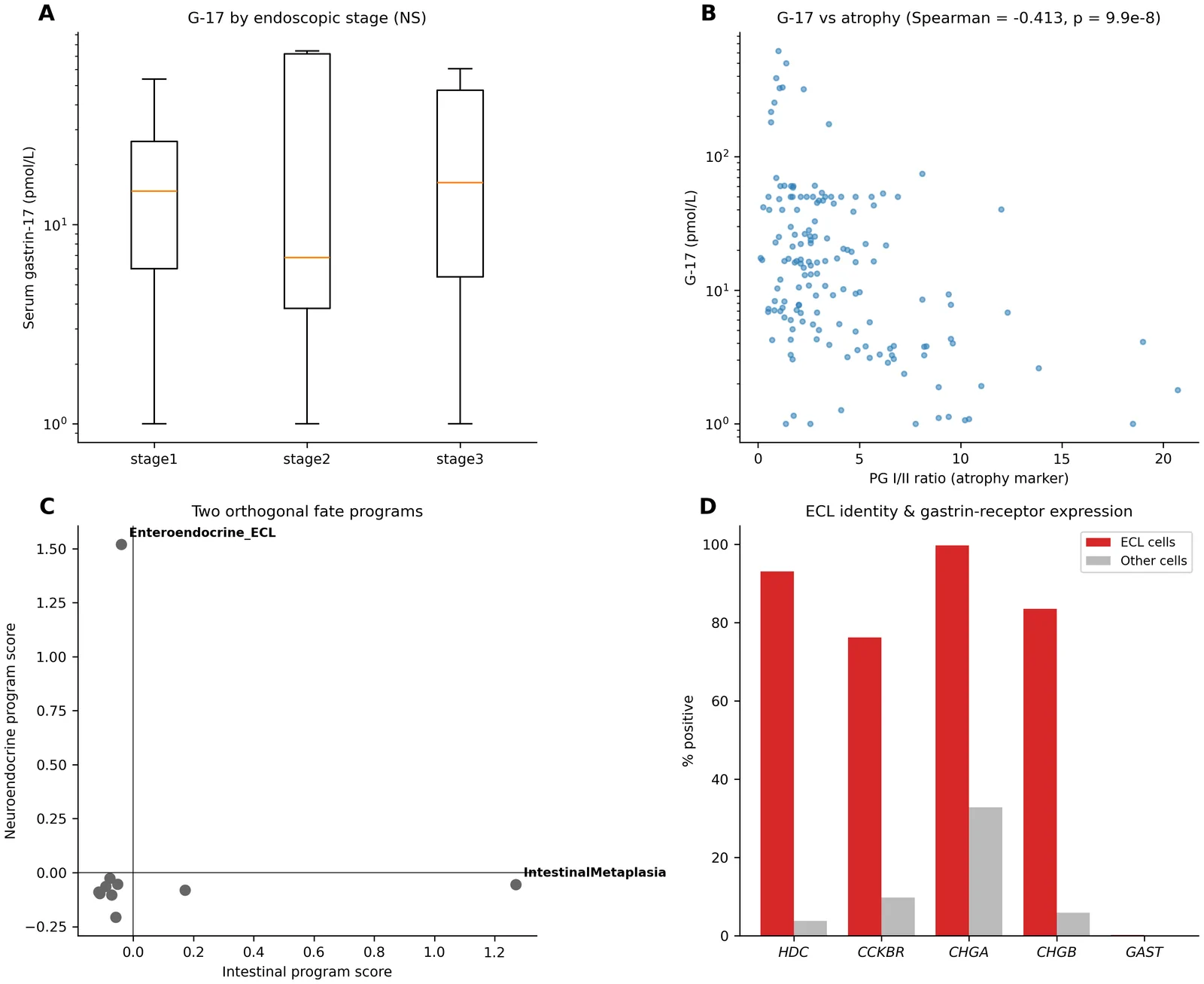
The serum-gastrin–ECL bridge and the divergent neuroendocrine fate. **(A)** Cohort serum G-17 by endoscopic stage (not significant). **(B)** G-17 scales inversely with the PG I/II ratio (*ρ* = −0.413, *p* = 9.9 × 10^−8^), i.e., with corpus atrophy. **(C)** The two fate programmes are orthogonal: intestinal metaplasia and ECL cells occupy opposite poles of the intestinal-versus-neuroendocrine plane. **(D)** ECL cells are *HDC*^+^ (93%) and *CCKBR*^+^ (76%; the gastrin receptor) but *GAST*-negative, versus all other cells.

The second fate departs from the glandular axis. We stress at the outset that the cohort serology (below) and the single-cell ECL data derive from *different* individuals, so this arm is a two-ended, mechanistic bridge rather than a within-patient correlation. In the clinical cohort, hypergastrinaemia was pervasive and tracked corpus atrophy: serum G-17 had a median of 15.6 pmol/L (IQR 5.0–42.2; range 1–1184; *n* = 156), exceeding 7 pmol/L in 66.7% and 30 pmol/L in 29.5% of patients, and correlated inversely with the PG I/II ratio (Spearman’s *ρ* = −0.413, *p* = 9.9×10^−8^, *n* = 154; **Figure 5**)—i.e., the more severe the corpus atrophy, the higher the gastrin, consistent with an achlorhydria-driven G-cell response. We report two honest negatives: G-17 was unrelated to endoscopic ROM stage (Spearman’s *ρ* = 0.04, *p* = 0.61; Kruskal–Wallis *p* = 0.88), and the single incident NET case had an unremarkable G-17 (7.8 pmol/L, 36th percentile; *n* = 1, uninterpretable).

At the tissue level, the two fates were molecularly orthogonal. Scoring the intestinal and neuroendocrine programmes per lineage placed intestinal metaplasia at one pole (intestinal 1.27, neuroendocrine −0.06) and ECL cells at the other (intestinal −0.04, neuroendocrine 1.52), with all other lineages near zero on both axes (**Figure 5**). The ECL population was a bona fide, gastrin-responsive ECL cell: it was 93.1% *HDC-*positive (ECL-specific; 3.8% elsewhere), 76.2% *CCKBR*-positive (the gastrin receptor; 9.8% elsewhere), 99.8% *CHGA*- and 83.5% *CHGB*-positive, and essentially *GAST*-negative (0.2%, as expected since ECL cells respond to but do not produce gastrin). Together, these data trace a coherent bridge: corpus atrophy → achlorhydria → hypergastrinaemia (cohort) → *CCKBR*^+^ ECL hyperplasia (single cell) → neuroendocrine fate — diverging from the intestinal-programme route to adenocarcinoma, yet sharing the same AIG parietal-cell–loss origin.

### 3.5 The immune microenvironment shows only weak, non-specific association with progression (exploratory)

Finally, we tested whether a cytotoxic microenvironment drives metaplastic progression along the cascade. The evidence was weak and is reported as exploratory. Immune cells were abundant (43%–55% across stages, myeloid-dominated), but their fraction did not increase monotonically with progression; at the sample level, the association with stage and with intestinal-metaplasia fraction was not significant (immune fraction versus stage ordinal *ρ* = 0.531, *p* = 0.062; versus metaplasia fraction *ρ* = 0.496, *p* = 0.085; *n* = 13). A cytotoxic programme did not track progression (peaking weakly at IMW, ≈ 0 elsewhere; cytotoxic versus metaplasia *ρ* = −0.25, versus pseudotime *ρ* = −0.30, both NS), *not* reproducing the CD8/cytotoxic enrichment seen in AIG-specific diagnostic studies—expected, since GSE134520 is a generic, largely antral cascade rather than AIG corpus tissue. The epithelial IFN-*γ* response was higher in the metaplastic mid-cascade and fell at EGC (NAG 0.16 → IMW 0.31 → EGC 0.11) and correlated only weakly with pseudotime (*ρ* = 0.237; the minuscule *p* ≈ 6.7×10^−249^ reflects the very large cell number, not a strong effect; **Supplementary Figure S1**). We therefore treat immune–progression coupling as a hypothesis requiring AIG-specific single-cell data with an immune compartment, not as a supported mechanism.

## 4 Discussion

Starting from a single autoimmune lesion—destruction of the gastric parietal cell—we have traced two divergent precancerous fates, one reconstructed molecularly end to end and one clinically and literature anchored, that share a common corpus origin. A single-cell map of the human AIG corpus fixed that origin, with near-absent parietal cells (0.45%) alongside substantial SPEM (18.6%), intestinal metaplasia (16.3%), and a distinct ECL population (3.4%). A molecular pseudotime built on an independent, histologically staged premalignant cascade recapitulated the morphological sequence (Spearman’s *ρ* = 0.496 between pseudotime and stage) and yielded an interpretable progression signature defined by a gastric-to-intestinal identity switch (up: *REG4*, *FABP1/2*, *ANPEP*, *KRT20*; down: *MUC5AC*, *GKN1/2*, *TFF2*, *PGC*). Transferring that signature back to the AIG corpus showed strong concordance with the AIG-intrinsic pseudotime (*ρ* = 0.673) and placed AIG metaplastic cells in the strong-metaplasia segment of the cascade (90.9% projected to IMS), establishing that *AIG enters the metaplasia-to-cancer axis from a corpus origin*. In parallel, the clinical cohort showed pervasive hypergastrinaemia that scaled with corpus atrophy (G-17 versus PG I/II ratio *ρ* = −0.413), and single-cell analysis identified a genuine, gastrin-responsive ECL population (*HDC*^+^ 93%, *CCKBR*^+^ 76%) whose neuroendocrine programme was orthogonal to the intestinal programme of the adenocarcinoma arm. The two fates are therefore molecularly separable yet developmentally linked through a shared AIG origin. To keep the strength of each claim explicit, **Table 3** separates what is directly shown by the data from what is inferred and what awaits future validation.

**Table 3:** Evidence level for the principal claims of the study, distinguishing what is directly shown by the data, what is inferred, and what requires future validation.

Finding/claim	Basis
Directly shown	
AIG corpus origin state: parietal-cell loss (0.45%), SPEM (18.6%), intestinal metaplasia (16.3%), ECL (3.4%)	GSE271866 scRNA (3 samples)
Cascade pseudotime increases with histological stage (*ρ* = 0.496)	GSE134520 scRNA
AIG progression score tracks AIG-intrinsic pseudotime (*ρ* = 0.673; up/down modules *ρ* = 0.34*/*−0.59)	Reference-based signature transfer
Hypergastrinaemia scales with corpus atrophy (G-17 vs. PG I/II ratio *ρ* =−0.413)	Clinical cohort (*n* ≈ 203)
ECL cells are gastrin-responsive (*HDC*^+^ 93%, *CCKBR*^+^ 76%)	GSE271866 scRNA
Intestinal and neuroendocrine programmes are molecularly separable	GSE271866 scRNA
Inferred	
AIG metaplastic cells map predominantly to strong metaplasia (90.9%)	Ingest projection; small subset (*n* = 55)
AIG occupies the atrophy-to-metaplasia segment of the adenocarcinoma axis	Relative orderings, not absolute levels
Serum-gastrin → ECL-hyperplasia route to the neuroendocrine fate	Two-ended bridge across different individuals
Future validation needed	
Molecular identity of the type-1 gastric NET endpoint	No transcriptomic data exist (reported gap)
Whether the immune microenvironment drives either fate	Exploratory, underpowered, non-AIG-specific
AIG-specific versus generic components of the signature	Needs an AIG-specific cascade with controls
Use for risk stratification or surveillance	Requires prospective, outcome-based cohorts

The separation makes explicit that the adenocarcinoma axis is molecularly reconstructed, whereas the neuroendocrine axis and any clinical-risk use remain inferential or aspirational.

### 4.1 Relation to existing work

These results give molecular substance to the previously conceptual framework of two distinct gastric cancer aetiologies (5). The progression signature is concordant with the established biology of SPEM and IM (9–15), but our reconstruction is anchored to a corpus, parietal-cell-loss origin rather than the *H. pylori*/antral Correa origin that dominates that literature (16, 17). To our knowledge, this is the first attempt to position AIG corpus epithelium on a metaplasia-to-cancer trajectory by reference-based transfer and, simultaneously, to articulate the divergent neuroendocrine fate in the same framework.

### 4.2 A general metaplastic programme that AIG enters from its own origin

A central interpretive question, raised directly by the reference data, is whether the progression signature reflects a *general* mucosal-injury response or one *specific* to autoimmune corpus atrophy. At the level of the gene programme, we read it as general: the signature captures a gastric-to-intestinal identity switch (loss of *MUC5AC*/*GKN1/2*/*TFF2*, gain of *REG4*/*FABP1/2* and *CDX2*-associated intestinal genes) that is shared across metaplastic injury of several aetiologies—which is precisely why it can be defined on a predominantly *H. pylori*/antral reference (GSE134520) and still transfer to the AIG corpus. AIG specificity therefore does not reside in the signature itself but in two places: the *origin* (a corpus, parietal-cell-loss starting point, rather than the antral *H. pylori* origin of the reference), and the demonstration that the same gastric-to-intestinal switch operates *within* AIG cells, where the up- and down-modules independently track the AIG-intrinsic pseudotime (*ρ* = 0.34 and −0.59). What our data cannot do is resolve, gene by gene, which elements of the module are uniquely autoimmune versus common to all gastric metaplasia; that distinction would require an AIG-specific premalignant cascade with matched non-AIG controls, which does not yet exist. We thus present the signature as a shared metaplastic programme that AIG demonstrably enters from its own origin, not as an AIG-private molecular route, and AIG-driven metaplasia need not follow the *H. pylori*/antral route in every detail.

### 4.3 Clinical implications

The serum-gastrin–ECL bridge has a directly translational reading. G-17 is already measurable, scaled with corpus atrophy in our cohort, and marks the trophic drive towards the ECL/neuroendocrine fate; coupled with the molecular progression signature and conventional severity markers (ROM stage, SPEM/IM status, and PG I/II ratio), it suggests a path towards a *severity-anchored, hypothesis-generating* framework that adds a molecular dimension on top of purely histological or serological staging such as OLGA and the corpus-predominant gastritis index (1, 2, 4, 18). We emphasise that this is a cross-sectional severity framing, not an outcome predictor: our cohort contains essentially no hard endpoints, so these findings do not by themselves justify any change to surveillance practice, and any risk stratifier built on these features would require prospective validation with nested feature selection.

### 4.4 Immunological implications

Because AIG is fundamentally an autoimmune disease, the immune compartment is a natural candidate driver of progression. Our exploratory analysis found the epithelial IFN-*γ* response elevated in the metaplastic mid-cascade, consistent with a contribution of interferon signalling to metaplastic conversion and with CD4 T-cell–driven (8) and IL-27–modulated (27) models of AIG carcinogenesis. The cytotoxic programme, however, did not track progression in this generic, largely antral cascade, so we explicitly refrain from a strong immune-mechanism claim: resolving how the autoimmune infiltrate steers the adenocarcinoma versus the neuroendocrine fate will require AIG-specific single-cell and spatial data that capture the immune–epithelial interface. We highlight this as the central immunological question that our framework raises.

### 4.5 The neuroendocrine molecular data gap as a finding

A central, deliberately reported result is the near-absence of public molecular data for the neuroendocrine endpoint: AIG-related ECL hyperplasia and type-1 gastric NET are essentially unprofiled transcriptionally (6, 7, 19). This gap forced the asymmetric design and is itself a call to action—the field needs dedicated expression and spatial profiling of ECL hyperplasia and type-1 gastric NET. In its absence, we bridged the fate with two-ended evidence (cohort gastrin plus a single-cell ECL programme) rather than a NET tumour profile, and we are explicit that we characterise the *route towards* the neuroendocrine fate, not the NET tumour itself.

### 4.6 Distinction from prior diagnostic work

This study is intentionally non-overlapping with earlier AIG transcriptomic work, including our own diagnostic discrimination of AIG versus *H. pylori*-associated atrophic gastritis. The question differs (precancerous progression versus diagnosis), the data substrate differs (a metaplastic single-cell cascade and an independent corpus map versus AIG diagnostic cohorts), the role of AIG data differs (an origin anchor versus the primary substrate), and the clinical data are used as a progression-severity reference rather than for diagnostic classification. No model, feature set, or claim is shared.

### 4.7 Strengths and limitations

The principal strengths are the explicit origin-to-cascade logic, the reference-based (rather than pooled) cross-dataset transfer that avoids a disease=batch confound, the use of marker-positive fractions to recover rare parietal and ECL states, and a transparent, honestly reported set of negatives. Several limitations temper the conclusions and should guide interpretation. (1) There is no molecular profile of the NET endpoint; the neuroendocrine arm rests on two-ended (cohort plus single-cell) and literature evidence, not on a NET tumour transcriptome. (2) The cohort has no matched tissue RNA, so the gastrin–tissue link is two-ended (not same-patient) and G-17 is correlated with atrophy markers rather than with molecular state directly. (3) The cascade reference (GSE134520) is a generic, largely antral premalignant series, not AIG-specific; AIG specificity is established by anchoring to the corpus map and by the transfer analysis, not assumed from the reference. (4) The AIG corpus data comprise only three samples with no matched normal control, and the AIG metaplasia subset is small (*n* = 55), so projected proportions, although robust in direction, need confirmation in larger AIG cohorts. (5) The AIG corpus pseudotime required pragmatic graph bridging to remain finite (the cascade did not, and a no-bridge sensitivity analysis preserved its stage ordering, *ρ* = 0.491); conclusions are framed as state coexistence and ordering rather than strict lineage causality. (6) Cross-dataset analysis used feature/label transfer (score_genes and ingest) rather than a joint embedding; a stronger integration (e.g., scANVI) is left for future work. (7) The immune arm is weak, non-AIG-specific, and underpowered at the sample level (*n* = 13), and the cytotoxic mechanism reported in AIG-specific diagnostic studies was not reproduced in this generic cascade; it should be revisited with AIG-specific single-cell or spatial data that include the immune compartment (8, 27). (8) G-17 was unrelated to endoscopic ROM stage, underscoring the known discordance between endoscopic and histological/serological atrophy, and the single incident NET case is uninterpretable. (9) The corpus dataset (GSE271866) has been used in prior diagnostic work; here, it is restricted to an origin-anchoring role for a distinct, progression-oriented question.

### 4.8 Future directions

The most consequential next steps are to generate AIG-specific single-cell and spatial transcriptomes that include immune and TCR information, to build the missing expression atlas of ECL hyperplasia and type-1 gastric NET, and to assemble an independent AIG cohort with matched histology, serum gastrin, paired tissue RNA, and longitudinal neoplastic outcomes. Such data would provide external validation, convert the two-ended gastrin–ECL bridge into a direct within-patient molecular link, enable a joint multi-dataset embedding, and allow prospective testing of whether the progression signature and the gastrin axis stratify precancerous risk along each fate.

## 5 Conclusion

By coupling public single-cell transcriptomes with a real AIG cohort, we trace how autoimmune gastritis diverges, from a shared corpus origin, towards two precancerous fates. The parietal-cell-loss corpus converges onto a metaplasia-to-cancer axis—the adenocarcinoma fate, reconstructed here at the molecular level—and, in parallel, atrophy-driven hypergastrinaemia couples to a *CCKBR*^+^ ECL programme that marks the neuroendocrine fate, which we anchor clinically and in the literature rather than reconstruct molecularly. The work delivers an interpretable progression signature, a serum-gastrin–ECL bridge that exploits a unique clinical asset, and an explicit accounting of the molecular data gap at the neuroendocrine endpoint. Framed as precancerous progression-risk biology and kept distinct from diagnostic discrimination, these findings provide a hypothesis-generating molecular scaffold for understanding AIG’s two precancerous fates; whether this scaffold can ultimately inform surveillance will require prospective, outcome-based validation.

## Data Availability

The datasets analysed for this study can be found in the NCBI Gene Expression Omnibus (GEO) under accession numbers GSE271866 (https://www.ncbi.nlm.nih.gov/geo/query/acc.cgi?acc=GSE271866) and GSE134520 (https://www.ncbi.nlm.nih.gov/geo/query/acc.cgi?acc=GSE134520). The analysis code and the de-identified clinical cohort data are available from the corresponding author on reasonable request, subject to institutional data-sharing policy.
