# Supplementary material for: Autoimmune gastritis diverges from a shared corpus origin: molecular reconstruction of the adenocarcinoma axis and clinical anchoring of the neuroendocrine axis
Source: Front Immunol. 2026 Aug 25;17:1899802. doi: 10.3389/fimmu.2026.1899802 (PMC13550166; doi:10.3389/fimmu.2026.1899802)
Supplement: Supplementary file 1 [file DataSheet1.pdf]

Supplementary Material

Autoimmune gastritis diverges from a shared corpus origin: molecular reconstruction of the adenocarcinoma axis and clinical anchoring of the neuroendocrine axis

Jingna Tao, Linghan Meng, Liju Zhang, Xiyan Zhang, Tong Li and Zhihong Li

Supplementary Table S1. Abbreviations

Abbreviation	Definition
AIG	autoimmune gastritis
SPEM	spasmolytic polypeptide-expressing metaplasia
IM	intestinal metaplasia
NET	neuroendocrine tumour
ECL	enterochromaffin-like (cell)
G-17	gastrin-17
PG	pepsinogen
PCA	parietal cell antibody
ROM	Royal Melbourne (endoscopic atrophy) stage
NAG	non-atrophic gastritis
CAG	chronic atrophic gastritis
IMW	weak intestinal metaplasia
IMS	strong intestinal metaplasia
EGC	early gastric cancer
DPT	diffusion pseudotime
HDC	histidine decarboxylase (ECL-specific)
CCKBR	cholecystokinin B receptor (the gastrin receptor)
GAST	gastrin (gene)
OLGA	Operative Link on Gastritis Assessment
scRNA-seq	single-cell RNA sequencing
UMAP	uniform manifold approximation and projection
IFN-γ	interferon-γ
TPOAb / TGAb	thyroid peroxidase / thyroglobulin antibody
GEO	Gene Expression Omnibus

Note on terminology: "pseudopyloric metaplasia" denotes the histological/endoscopic finding and "SPEM" the corresponding single-cell state; we treat them as the same metaplastic programme observed at different levels of resolution.

Supplementary Figure S1

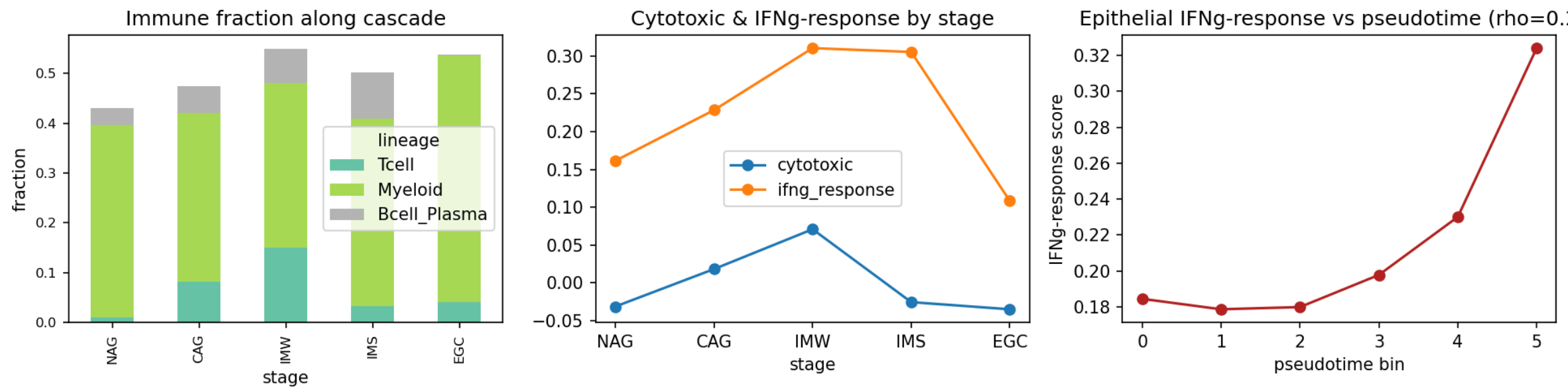

Figure S1: Exploratory immune-microenvironment analysis on the premalignant cascade (GSE134520). Left: immune-compartment fraction by stage; middle: cytotoxic and IFN-γ response scores by stage; right: epithelial IFN-γ response versus pseudotime. Associations with progression are weak and largely non-significant at the sample level ($n = 13$); this arm is reported as exploratory and does not, on its own, support an immune-driven mechanism for either fate.
